# An Inductive Logistic Matrix Factorization Model for Predicting Drug-Metabolite Association With Vicus Regularization

**DOI:** 10.3389/fmicb.2021.650366

**Published:** 2021-04-01

**Authors:** Yuanyuan Ma, Lifang Liu, Qianjun Chen, Yingjun Ma

**Affiliations:** ^1^School of Computer and Information Engineering, Anyang Normal University, Anyang, China; ^2^School of Education, Anyang Normal University, Anyang, China; ^3^School of Computer, Central China Normal University, Wuhan, China; ^4^School of Applied Mathematics, Xiamen University of Technology, Xiamen, China

**Keywords:** logistic matrix factorization, drug-metabolite association, Vicus matrix, human metabolites, graph regularization

## Abstract

Metabolites are closely related to human disease. The interaction between metabolites and drugs has drawn increasing attention in the field of pharmacomicrobiomics. However, only a small portion of the drug-metabolite interactions were experimentally observed due to the fact that experimental validation is labor-intensive, costly, and time-consuming. Although a few computational approaches have been proposed to predict latent associations for various bipartite networks, such as miRNA-disease, drug-target interaction networks, and so on, to our best knowledge the associations between drugs and metabolites have not been reported on a large scale. In this study, we propose a novel algorithm, namely inductive logistic matrix factorization (ILMF) to predict the latent associations between drugs and metabolites. Specifically, the proposed ILMF integrates drug–drug interaction, metabolite–metabolite interaction, and drug-metabolite interaction into this framework, to model the probability that a drug would interact with a metabolite. Moreover, we exploit inductive matrix completion to guide the learning of projection matrices *U* and *V* that depend on the low-dimensional feature representation matrices of drugs and metabolites: *F^m^* and *F^d^*. These two matrices can be obtained by fusing multiple data sources. Thus, *F^d^U* and *F^m^V* can be viewed as drug-specific and metabolite-specific latent representations, different from classical LMF. Furthermore, we utilize the Vicus spectral matrix that reveals the refined local geometrical structure inherent in the original data to encode the relationships between drugs and metabolites. Extensive experiments are conducted on a manually curated “DrugMetaboliteAtlas” dataset. The experimental results show that ILMF can achieve competitive performance compared with other state-of-the-art approaches, which demonstrates its effectiveness in predicting potential drug-metabolite associations.

## Introduction

With the development of metabolomics technology, more and more metabolites have been identified. This progress provides unprecedented opportunities to obtain new insights into the effects of drugs on metabolites. Recently, Liu et al. (2020) integrated epidemiologic, pharmacologic, genetic, and gut microbiome data to analyze the relationships between drugs and metabolites, which provided a trail for targeted experimental pharmaceutical research to improve drug safety and efficacy. Exploring the potential drug-metabolite associations is also a novel route towards pharmacomicrobiomics and precision medicine. Doestzada et al. (2018) reviewed the complex interactions between host, intestinal microorganisms and drugs, and thought that pharmacomicrobiomics would provide an important foundation for personalized medicine and precision medicine. The earliest report about interactions between drugs and metabolites can be dated back to the 1930s with the discovery of sulphanilamide (Fuller, 1937). The activity of prontosil is due to the transformation of microbial azoreductases and the liberation of sulphanilamide. In addition, microbial metabolites can also inactivate drugs, such as digoxin. A study on *Eggerthella lenta* strains in 2013 (Haiser et al., 2013) found that these strains carried a two-gene cardiac glycoside reductase (cgr) operon that was transcriptionally activated by digoxin (Doestzada et al., 2018), and thus resulted in the inactivation of the drug in cardiovascular treatment.

Identifying drug-metabolite associations not only provides deep insights into understanding complex interaction mechanisms among them, but it can also benefit the screening of chemical compounds for drug development and improve microbe related therapy. The complex relationship between drugs, metabolites, and microbes has attracted extensive attention. However, conventional wet-lab research for verifying drug-metabolite interactions is generally labor-intensive, costly, and time-consuming. Computational approaches are a viable alternative. Shang et al. (2014) found that metabolites in the same pathway were usually associated with the similar or same disease. Based on this fact, they proposed a metabolite pathway-based random walk algorithm to prioritize the candidate disease metabolites (Shang et al., 2014). Yao et al. (2015) presented an approach based on global distance similarity to predict and prioritize disease related metabolites. Ma et al. (2020b) integrated multiple diseases and metabolite similarity networks to predict the potential associations between metabolites and diseases. Long and Luo (2020) used multi-source biomedical data to construct a three-level heterogeneous network and designed a novel network embedding representation framework to identify microbe-drug associations. Specifically, Long et al. (2020) exploited the conditional random field, graph convolutional network and a random walk with restart (RWR) to learn the latent feature representations of drugs and microbes, identifying some potential drug-microbe associations.

Although these studies have obtained some valuable results, there are two main limitations to the existing drug-microbe or metabolite-disease association mining approaches. Firstly, the accuracy of these methods is still unsatisfactory due to a lack of sufficient prior information for drugs, microbes, and diseases. Secondly, the local geometrical structure of nodes is important in the task of dimensionality deduction and data representation, which decides the effectiveness and efficiency of algorithms to a large extent. The algorithms mentioned above did not consider the local spectral information that resides in the original data, meaning their performances are not ideal.

**FIGURE 1:**
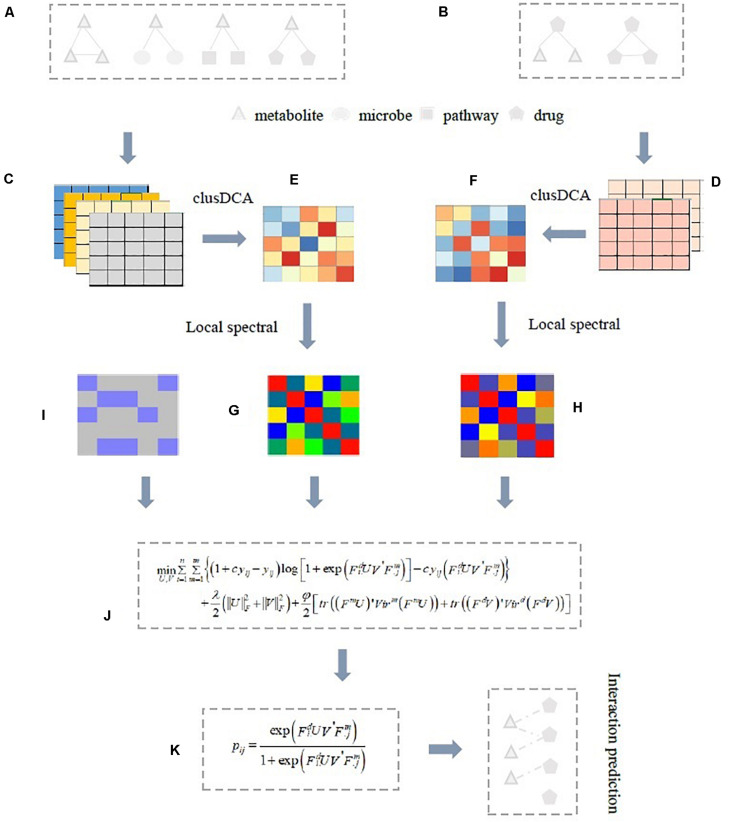
Illustrative example of ILMF for predicting potential drug-metabolite associations. **(A)** Metabolite–metabolite, metabolite-drug, metabolite-microbe, metabolite-pathway association matrices, or correlation matrices; **(B)** Drug-metabolite, drug–drug association, or correlation matrices; **(C,D)** Based on Gaussian interaction profile kernel function, metabolite–metabolite similarity matrices, and drug–drug similarity matrices obtained from four metabolite association data and two drug association data, respectively; **(E)** The fused metabolite–metabolite similarity matrix by integrating four metabolite-related data with clusDCA; **(F)** The fused drug–drug similarity matrix by integrating two drug association data with clusDCA. Then, the local spectral matrix of metabolites **(G)** And the local spectral matrix of drugs **(H)** Can be obtained based on these two fused similarity matrices with Vicus; **(I)** The drug-metabolite association matrix; **(J)** The proposed ILMF model. Finally, ILMF outputs the predicted drug-metabolite interaction probability scores **(K)**. Here, a solid line indicates known associations, a dotted line indicates predicted drug-metabolite associations obtained from ILMF.

In this study, we propose a novel computational approach, named inductive logistic matrix factorization (ILMF), to analyze latent drug-metabolite associations. ILMF integrates the advantages of logistic matrix factorization (LMF; Johnson, 2014; Liu et al., 2016) and inductive matrix completion (Natarajan and Dhillon, 2014; Chen et al., 2018) to learn low-dimensional embedding of drugs and metabolites, and predict the final interaction probabilities based on the two low-dimensional representation of drugs and metabolites. Specifically, ILMF first learns the latent representation of drugs and metabolites via clusDCA (Cho et al., 2015; Wang et al., 2015), which runs RWR on each node in each interaction network (e.g., metabolite–metabolite interaction network or similarity network) to compute “the diffusion state” of each point, and then utilizes a singular value decomposition (SVD)-based approach to obtain the consensus low-dimensional matrix representation for metabolites and drugs *Z*^*m*^ and *Z*^*d*^, respectively. Secondly, based on *Z*^*m*^ and *Z*^*d*^, ILMF exploits LMF to learn two projection matrices *U* and *V*, respectively, so that *Z^*m*^V* and *Z^*d*^U* have the same semantic space. Finally, a logistic function is used to predict the probability that a drug would interact with a metabolite in the same way that LMF does. Nevertheless, in contrast to LMF, ILMF captures the topological properties of nodes (i.e., drugs or metabolites) and takes advantage of the idea of inductive matrix completion (Luo et al., 2017) to generate the optimal projection of drugs and metabolites. In addition, ILMF also exploits the local spectral Vicus matrices (Wang B. et al., 2017) of drugs and metabolites to reveal the refined local geometrical structure inherent in drug–drug interaction network and metabolite–metabolite interaction network. An illustrative example of this pipeline is given in Figure 1, followed by a more detailed description of ILMF in section “Materials and Methods.”

The contributions of this article are summarized as follows:

1. We propose a novel LMF-based framework, named ILMF, to predict drug-metabolite associations by integrating multiple biological networks. To the best of our knowledge, this is the first work to predict the latent drug-metabolite associations.
2. ILMF combines the advantages of inductive matrix completion and the local spectral Vicus matrix of each interaction network into this framework, and captures the optimal low-dimensional representation of drugs and metabolites.
3. We have manually curated a drug-metabolite association dataset (“DrugMetaboliteAtlas”) by retrieving relevant literature. This benchmark dataset can be used to evaluate the performance of various association prediction algorithms, which facilitates future research in drug-metabolite association prediction tasks.

The comprehensive experiments show that the proposed ILMF algorithm outperforms several state-of-the-art methods on the curated “DrugMetaboliteAtlas” dataset. In addition, the prediction ability of ILMF has also been confirmed by retrieving the latest published literature or information from databases.

## Materials and Methods

### Materials

The “DrugMetaboliteAtlas” dataset was downloaded from the BBRMI-NL website^1^ (Liu et al., 2020). It contains 1071 interactions from 87 commonly prescribed drugs and 150 clinically relevant metabolites. After removing drugs lacking significantly relevant metabolite associations, 42 drugs were reserved. In addition, we also manually curated the correlations between drug categories and the correlations between metabolites in the Rotterdam study (Liu et al., 2020).

Metabolite-microbe associations and metabolite-pathway associations were also downloaded from literature (Kurilshikov et al., 2019). The metabolite similarities from each type of association were computed based on the Gaussian interaction profile kernel (He et al., 2018; Ma et al., 2020b). After that, clusDCA (Wang et al., 2015) was used to fuse multiple drug–drug interaction networks and multiple metabolite–metabolite interaction networks. Simultaneously, the optimal low-dimensional matrix representations of metabolites and drugs *F^m^*, *F^d^* can also be obtained from this fusing process. Then, the local Vicus spectral matrices of metabolites and drugs *V*^*irm*^, *V*^*ird*^ were computed based on the optimal low-dimensional matrix representations of metabolites and drugs *F^m^* and *F^d^*, respectively. Finally, the low-dimensional feature matrices of drugs and metabolites *F^m^* and *F^d^*, the local spectral matrices *Vir^m^* and *Vir^d^* were used as input of the proposed ILMF algorithm.

### Problem Formalization

In this article, the set of drugs is denoted by $D={\left\{d_{i}\right\}}_{i=1}^{n}$, and the set of metabolites is denoted by $M={\left\{m_{j}\right\}}_{j=1}^{m}$, where, *n* and *m* are the number of drugs and metabolites, respectively. The known drug-metabolite interactions are represented as a *n*×*m* binary matrix *Y* ∈ *R*^*n*×*m*^, where *y*_*ij*_ = 1 if a drug *d*_*i*_ has been observed to interact with a metabolite *m*_*j*_; otherwise *y*_*ij*_ = 0.

This study aimed to solve the problem of predicting the interaction probability of a drug-metabolite interaction pair, and subsequently rank the candidate drug-metabolite pairs based on these probabilities in descending order. Thus, the top-ranked pairs can be viewed as the most relevant interactions.

### Metabolite–Metabolite Similarity

There are four metabolite related data sources: metabolite–metabolite correlation matrix *Cor*_*m*_, metabolite-microbial species association matrix *MM*, metabolite-pathway association matrix *MP*, and drug-metabolite interaction matrix *Y*. *Cor*_*m*_ is obtained from literature (Liu et al., 2020); *MM* and *MP* are collected from literature (Kurilshikov et al., 2019).

For drug-metabolite association matrix *Y*, we use the Gaussian interaction profile kernel (He et al., 2018) to compute the similarity between any two metabolites. Let the *j*-th column *y*_.*j*_ of *Y* denote the interaction profile between metabolite *m*_*j*_ and all drugs. For any two metabolites *m*_*i*_ and *m*_*j*_, the similarity between them can be measured as:

(1) $$K_{m\,d}=\exp\left(-\gamma_{m}\,{||y_{\cdot i}-y_{\cdot j}||}^{2}\right).$$

Where γ_*m*_ is a bandwidth parameter that needs to be normalized based on a new bandwidth parameter $\gamma_{m}^{\prime }$ :

(2) $$\gamma_{m}=\gamma_{m}^{\prime }\,/\,\left(\frac{1}{m}\,\sum_{l=1}^{m}{\left|y_{\cdot l}\right|}^{2}\right).$$

Here, *m* is the number of metabolites. |⋅| denotes Frobenius norm. $\gamma_{m}^{\prime }$ is set to be 1 according to the previous study (Wang F. et al., 2017; He et al., 2018).

The Gaussian profile kernel similarity matrices *K*_*mm*_ and *K*_*mp*_ can also be computed based on metabolite-microbial species association matrix *MM* and metabolite-pathway association matrix *MP*, respectively.

### Drug–Drug Similarity

There are two drug related data sources: drug–drug correlation matrix *Cor*_*d*_ and drug-metabolite interaction matrix *Y*. *Cor*_*d*_, which were obtained from literature (Liu et al., 2020). Analogously, the Gaussian interaction profile kernel similarity matrix *K*_*d*_ between any two drugs can be computed in the same way.

After obtaining four metabolite–metabolite similarity matrices and two drug–drug similarity matrices derived from multiple data sources, we used clusDCA (Cho et al., 2015; Wang et al., 2015) to fuse these similarity matrices and finally acquire the optimal low-dimensional matrix representations of metabolite and drug features *F^m^* and *F^d^*, respectively.

### Inductive Logistic Matrix Factorization

Logistic matrix factorization has been demonstrated to be effective in the prediction of drug-target interactions (Liu et al., 2016), metabolite-disease (Ma et al., 2020a), and personalized recommendations (Hu et al., 2008; Johnson, 2014; Liu et al., 2014). The main advantage of LMF is that it assigns higher levels of importance to the observed interaction pairs than unknown ones. In this study, we apply LMF for drug-metabolite interaction prediction. LMF maps drugs and metabolites into a shared low-dimensionality latent semantic space *r*≪*min*⁡(*m*, *n*). The interaction probability *p*_*ij*_ of a drug-metabolite pair (*d*_*i*_,*m*_*j*_) can be modeled as follows:

(3) $$p_{i\,j}=\frac{\exp\left(w_{i}\,h_{j}^{\prime }\right)}{1+\exp\left(w_{i}\,h_{j}^{\prime }\right)}.$$

Where *w*_*i*_ ∈ *R*^1×*r*^, *h*_*j*_ ∈ *R*^1×*r*^ are latent representations of drug *d*_*i*_ and metabolite *m*_*j*_, respectively. For convenience, we further represent the latent vectors of all drugs and metabolites as matrix form *W* ∈ *R*^*n*×*r*^ and *H* ∈ *R*^*m*×*r*^, respectively.

The observed drug-metabolite interaction pairs are generally more reliable and important than the unknown interaction pairs. A higher level of importance was thus assigned to known interaction pairs than unknown ones. According to a previous study, we set the importance level to be *c*(*c*≥1). Eq. 3 can be written as follows:

(4) $$p\,\left(Y|U,V\right)=\prod_{1\leq i\leq n,\,1\leq j\leq m,y_{i\,j}=1}{\left[p_{i\,j}^{y_{i\,j}}\,{\left(1-p_{i\,j}\right)}^{\left(1-{\text{y}}_{i\,j}\right)}\right]}^{c}\times \prod_{1\leq i\leq n,\,1\leq j\leq m,y_{i\,j}=0}{\left[p_{i\,j}^{y_{i\,j}}\,{\left(1-p_{i\,j}\right)}^{\left(1-{\text{y}}_{i\,j}\right)}\right]}^{.}$$

Here, *c* is the important level parameter used to control the weight assigned to the observed drug-metabolite pairs. In the next experiments, we empirically set it to two.

Inspired by the ideas of inductive matrix completion (Jain and Dhillon, 2013; Zeng et al., 2020) and generalized matrix factorization (GMF) (Zhang et al., 2020), we designed a novel ILMF framework, ILMF, to predict the latent interaction probabilities between drugs and metabolites. In particular, we used *F^d^* ∈ *R*^*n*×*k*_1_^ and *F^m^* ∈ *R*^*m*×*k*_2_^ derived from clusDCA (see section “Drug–Drug Similarity”) to guide the learning process of projection matrices *U* ∈ *R*^*k*_1_×*r*^ and *V* ∈ *R*^*k*_2_×*r*^, so that the latent representations of metabolites and drugs *W* = *F^d^U* ∈ *R*^*n*×*r*^ and *H* = *F^m^V* ∈ *R*^*m*×*r*^ can carry compatible and complementary information from multiple data sources. Thus, in the ILMF model, Eq. 3 can be rewritten as follows:

(5) $$p_{i\,j}=\frac{\exp\left(F_{i.}^{d}\,U\,V^{\prime }\,F_{.j}^{m}\right)}{1+\exp\left(F_{i.}^{d}\,U\,V^{\prime }\,F_{.j}^{m}\right)}.$$

Where $F_{i.}^{d}$ denotes the *i*-th row of *F^d^*, $F_{.j}^{m}$ denotes the *j*-th column of *F^m^*. By substituting Eq. 5 into Eq. 4, we estimate the projection matrices *U* and *V* by maximizing the above likelihood function (Eq. 3), which is equivalent to minimizing the negative logarithm of Eq. 3. Thus, the objective function of the proposed ILMF framework can be defined as:

(6) $$\min_{U,V}\,\sum_{i=1}^{n}\sum_{m=1}^{m}\left(1+c\,y_{i\,j}\,y_{i\,j}-y_{i\,j}\right)\,\log\left[1+\exp\left(F_{i.}^{d}\,U\,V^{\prime }\,F_{.j}^{m}\right)\right]-CY_{ij}\left(F_{i}^{d}UV'\text{​}F_{j}^{m}\right).$$

To avoid overfitting, the L_2_ regularization is generally imposed on *U* and *V*. Thus, Eq. 6 becomes:

(7) $$\min_{U,V}\sum_{i=1}^{n}\sum_{m=1}^{m}\{\left(1+cy_{i\,j}-y_{i\,j}\right)\log\left[1+\exp\left(F_{i.}^{d}UV^{\prime }F_{.j}^{m}\right)\right]-CY_{ij}\;\left(F_{i}^{d}UV'\text{​}F_{j}^{m}\right)\}+\frac{\lambda }{2}\left(|U|\frac{2}{F}+|V|\frac{2}{F}\right),$$

Where λ is a regularization parameter used to tradeoff the balance between reconstruction errors and smooth solutions.

Note that, for new drugs (metabolites) that do not have any known connections with metabolites (drugs), ILMF can still predict their potential associations, once we get their similarity network from other data sources. This is different from GMF (Zhang et al., 2020). In GMF, the neighborhood information of nodes was used to generate two feature matrices, and then they were adaptively updated at each iteration. In contrast, ILMF fuses multiple similarity networks to produce the low-dimensional matrix representations of metabolites and drugs.

### Vicus Matrix

As demonstrated in literature (Wang B. et al., 2017), Vicus has many of the same properties as Laplacian. However, compared with Laplacian, Vicus can capture the local geometrical structure that resides within the original data well. The reason for using Vicus instead of Laplacian is that the local connection information from neighboring nodes makes the learned graph more robust to noise and helps to alleviate the influence of outliers.

Let {*x*_1_,*x*_2_,…,*x*_*n*_} be the set of data points. Corresponding to *x*_*i*_, *v*_*i*_ denotes the *i*-th vertex in a weighted network *P*, and *N*(*i*) represents *x*_*i*_ ’s neighborhood, not including *x*_*i*_. Here, the neighborhood size of all nodes is consistent (_— N_i — =k,  i=1,2,…,n_).

Based on the assumption that the cluster label of the *i*-th data point can be inferred from its nearest neighborhood *N*(*i*), we first extract a subnetwork *P*_*i*_ = (*V*_*i*_*E*_*i*_) such that V*_i_* = *N*(*i*)⋃*x*_*i*_. *E*_*i*_ represents the edges connecting all points in *V*_*i*_. Using the label diffusion algorithm (Zhou et al., 2004), a virtual label indicator vector $c_{V_{i}}^{k}$ can be reconstructed as:

(8) $$c_{v_{i}}^{k}=\left(1-\alpha \right)\,{\left(I-\alpha \,S_{i}\right)}^{-1}\,q_{v_{i}}^{k},1\leq k\leq C.$$

Where α ∈ (0, 1) is a constant, *C* is the number of clusters, $q_{V_{i}}^{k}$ is the scaled cluster indicator of *P*_*i*_. *S*_*i*_ denotes the normalized transition matrix, i.e., $S_{i}\,\left(u,t\right)=P_{i}\,\left(u,t\right)\,/\,\sum_{l=1}^{K+1}P_{i}\,\left(u,l\right)$. $c_{V_{i}}^{K}$ is a vector including *K* + 1 elements. Here, ${\bar{q}}_{i}^{k}=c_{V_{i}}^{k}\,\left[K+1\right]$ is the estimate of how likely it is that node *i* belongs to the *k*-th cluster. The goal is to maximize the concordance between ${\bar{q}}_{i}^{k}$ and $q_{i}^{k}$. Let β_*i*_ ∈ *R*^*K* + 1^ be the *i-th* row of the matrix (1−α)(*I*−_α_*S*_*i*_)−1, representing label propagation at its terminal state. We set ${\bar{q}}_{i}^{k}=\beta_{i}\,q_{V_{i}}^{k}$. Thus, ${\bar{q}}_{i}^{k}$ can be approximated to:

(9) $${\bar{q}}_{i}^{k}\approx \frac{\beta_{i}\left[1:K\right]q_{N\,\left(i\right)}^{k}}{1-\beta_{i}\,\left[K+1\right]}.$$

Where β_*i*_[1:*K*] denotes the first *K* elements of β_*i*_ and β_*i*_[*K* + 1]denotes the (*K* + 1)*-*th element in β_*i*_.

Next, we used matrix *B* to represent the linear relationship: ${\bar{q}}^{k}\approx B\,q^{k},k=1,2,\ldots ,C$:

(10) $$B_{i\,j}=\left\{ \begin{array}{cc} \frac{\beta_{i}\,\left[j\right]}{1-\beta_{i}\,\left[K+1\right]} & \text{if}\,x_{j}\in N\,\left(i\right)\,\text{and}\,x_{j}\,\mathrm{is}\,\mathrm{the}\,j-\mathrm{th}\,\mathrm{element}\,\mathrm{in}\,N\,\left(i\right);\\ 0 & \text{otherwise} \end{array}\right.$$

To minimize the difference between ${\bar{q}}^{k}$ and *q^k^*, an objective function can be defined as follows:

(11) $$\sum_{i=1}^{n}\sum_{k=1}^{C}{\left({\bar{q}}_{i}^{k}-q_{i}^{k}\right)}^{2}=\sum_{k=1}^{C}{\left|{\bar{q}}^{k}-q^{k}\right|}^{2}\approx \sum_{k=1}^{C}{\left|q^{k}-B\,q^{k}\right|}^{2}=T\,r\,\left(Q^{T}\,{\left(I-B\right)}^{T}\,\left(I-B\right)\,Q\right).$$

Here, *Tr*(•) denotes the trace of a matrix. Setting *Vir* = (*I*−*B*)^*T*^(*I*−*B*), we thus obtain the Vicus matrix. In this study, we propose to exploit the Vicus matrix as a graph regularization term to enhance the prediction performance of ILMF.

Note that each item in the Vicus matrix obtained from Eq. 11 represents the probability of vertex *i* having the same label as vertex *j*. Encoding the local neighborhood of each vertex in this way does not only preserve the geometric attributes of the Laplacian matrix but also improves the quality of clustering (Nelson et al., 2019). Wang B. et al. (2017) indicated the Vicus-based spectral clustering approach outperformed Laplacian-based methods on many biological tasks, such as single-cell RNA data clustering, recognition of rare cell populations, the ranking of genes related to cancer subtypes and so on. Therefore, in this manuscript, we use Vicus spectral matrix to model fine-grained connections between drugs and metabolites.

### Vicus Regularization Based Inductive Logistic Matrix Factorization

The final drug-metabolite association prediction model can be constructed by considering the existing drug-metabolite links and the local geometrical structure of drugs and metabolites. By introducing Vicus regularization into Eq. 7, the proposed ILMF method is formulated as follows:

(12) minU,V∑i=1n∑m=1m{(1+cyi⁢j-yi⁢j)log[1+exp(Fi.dUV′F.jm)]−cyij (FidUV​'​Fjm)}+λ2(|U|2F+|V|2F)+ϕ2[tr((FmU)'​Virm(FmU))+tr((FdV)'Vird(FdV))].

Where ϕ is a graph regularization parameter. *Vir^m^* is the Vicus matrix of metabolites, and *Vir^d^* is the Vicus matrix of drugs. Note that, in this study, we exploit the cosine similarity of the low-dimensional feature matrix of metabolites *F^m^* (or drugs_*F^d*_) to compute the Vicus matrix *Vir^m^* or *Vir^d^*, respectively.

The optimization problem in Eq. 12 can be solved by an alternating gradient ascent scheme. In particular, we adopt the AdaGrad algorithm (Duchi et al., 2011) to update *U* and *V*. Further details can be found in the study by Liu et al. (2016). Once the projection matrices *U* and *V* have been obtained, the association probability of any drug-metabolite pair can be predicted by Eq. 5. However, for many unobserved interaction pairs, the learned latent representation of drugs and metabolites may not be accurate since they are only based on unknown drug-metabolite pairs.

To address this problem, we adopted the practices outlined in other literature (Ma et al., 2020a). Let ${\text{N}}_{d}^{+}=\left\{m_{i}|\sum_{j}y_{i\,j}>0\right\}$ and $N_{m}^{+}=\left\{m_{j}|\sum_{i}y_{i\,j}>0\right\}$ denote the sets of observed drugs and metabolites, respectively. $N_{d}^{+}\,\left(d_{i}\right)$ denotes the set of *K* nearest neighbors of *d*_*i*_ in $N_{d}^{+}$. Similarly, $N_{m}^{+}\,\left(m_{j}\right)$ denotes the set of *K* nearest neighbors of *m*_*j*_ in $N_{m}^{+}$. We can replace the latent vector representation of a drug or metabolite with the representations of its neighbors. Then, for each drug *d*_*i*_, the revised ${\bar{w}}_{i}$ is defined as:

(13) $${\bar{w}}_{i}=\left\{ \begin{array}{cc} w_{i}, & \text{if}\;d_{i}\in N_{d}^{+}\\ \frac{1}{Q_{i}^{d}}\,\sum_{l=1}^{K}\mu_{l}^{d}\,w_{l}, & \text{if}\;d_{i}\notin N_{d}^{+} \end{array}\right..$$

Where $Q_{i}^{d}=\sum_{l=1}^{K}\alpha^{l-1}\,S^{d}\,\left(d_{i},d_{l}\,d_{l}\right)$ is a normalized term, *S^d^* = *cosine*(*F^d^*,*F^d^*) denotes the consensus drug–drug similarity matrix derived from multiple similarity networks. *d*_*l*_ indicates the *l*-th neighbor in $N_{d}^{+}\,\left(d_{i}\right)$ sorted in descending order according to the similarity with *d*_*i*_. α ∈ [0, 1] is a decay factor, and $\mu_{l}^{d}=\alpha^{l-1}\,S^{d}\,\left(d_{i},d_{l}\right)$ is a weight factor. Similarly, we can also obtain the optimal latent representation ${\bar{m}}_{j}$ for each metabolite *m*_*j*_ :

(14) $${\bar{h}}_{j}=\left\{ \begin{array}{cc} h_{j}, & \text{if}\,m_{j}\in N_{m}^{+}\\ \frac{1}{Q_{i}^{m}}\,\sum_{l=1}^{K}\mu_{l}^{m}\,h_{l}, & \text{if}\,m_{j}\notin N_{m}^{+} \end{array}\right..$$

Where $Q_{j}^{m}=\sum_{l=1}^{K}\alpha^{l-1}\,S^{m}\,\left(m_{j},m_{l}\right)$, *S^m^* = *cosine*(*F^m^*,*F^m^*) indicates the consensus metabolite–metabolite similarity matrix. *m*_*l*_ is the *l*-th neighbor in $N_{m}^{+}\,\left(m_{j}\right)$, which is sorted in descending order according to similarity with *m*_*j*_. $\mu_{l}^{m}=\alpha^{l-1}\,S^{m}\,\left(m_{j},m_{l}\right)$ is a weight factor.

Finally, the interaction probability of a drug-metabolite pair is redefined as follows:

(15) $${\bar{p}}_{i\,j}=\frac{\exp\left({\bar{w}}_{i}\,{\bar{h^{\prime }}}_{j}\right)}{1+\exp\left({\bar{w}}_{i}\,{\bar{h^{\prime }}}_{j}\right)}.$$

To demonstrate the flowchart of ILMF, the pseudocode of ILMF is given in Table 1.

**TABLE 1:** The pseudocode of the ILMF algorithm.

Input: The known association matrix *Y*; parameters λ, ϕ, *c*, *K*
Output: The projection matrices, *U* and *V*
1. Compute metabolite–metabolite similarity matrices *K*_*md*_, *K*_*mm*_, *K*_*mp*_ according to Eqs 1 and 2, respectively; similarly, compute drug–drug similarity matrices *K*_*d*_;
2. Compute the low-dimensional feature representational matrices of metabolites and drugs, *F*^*m*^ and *F*^*d*^ using clusDCA (Cho et al., 2015); computing Vicus spectral matrices of metabolites and drugs, *Vir*^*m*^ and *Vir*^*d*^;
3. Initialize *U* and *V* randomly;
4. For *t = 1,……, max_iter* do
5. Update *U* and *V* according to AdaGrad algorithm
6. Until convergence conditions are satisfied
7. End for
8. Return *U, V*

## Results and Discussion

### Experimental Settings

Following the previous studies (Zheng et al., 2013; Ding et al., 2014; Liu et al., 2016; Zhang et al., 2018a,b, 2019, 2020; Ma et al., 2020a), the performance of various association prediction methods can be evaluated by performing fivefold cross-validation (CV). For each method, we perform fivefold CV five times. Then, we calculate the area under the receiver operating characteristic curve (AUC), the area under the precision-recall curve (AUPR) scores in each repetition of CV, and the final AUC and AUPR scores are obtained by calculating the average over the five repetitions.

The object of this study is to predict the latent drug-metabolite associations. For the known drug-metabolite interaction matrix *Y* ∈ *R*^*n*×*m*^ with *n* drugs and *m* metabolites, we conduct CV on randomly selected drug-metabolite pairs. Specifically, we randomly divide the observed and unobserved interaction pairs into five equal parts. Then, in each round, one is used as test data, the remaining entries in *Y* are used for training. Thus, each of the five test datasets (or training data) includes the same number of observed and unobserved interaction pairs.

Note that we do not consider the other two scenarios for CV experiments: random rows or columns selected for testing. It is mainly because the drug-metabolite association matrix is commonly sparse, and the drug–drug or metabolite–metabolite similarity information from external sources cannot provide enough aid for prediction.

### Evaluation Metrics and Competing Approaches

In this study, the AUC, AUPR, and F1 value are used as the evaluation metrics. These metrics have been widely used in various association prediction tasks. To demonstrate the effectiveness and efficiency of our proposed ILMF algorithm in predicting drug-metabolite interaction, we compare the proposed ILMF method with the following several state-of-the-art approaches, namely, DTInet (Luo et al., 2017), IMCMDA (Chen et al., 2018) and GRNMF (Xiao et al., 2018). These approaches were originally designed for DTI prediction or miRNA-disease association prediction. Furthermore, we can obtain a variant of ILMF, which learns *U* and *V* with the consensus similarity matrices of drugs and metabolites instead of their Vicus matrices. Here, we denote this variant as ILMF^–^, which has a similar objective function to MNLMF (Ma et al., 2020a) and NRLMF (Liu et al., 2016).

For all the compared methods above, their performance is reported with best-tuned parameters.

### Experimental Results

In this subsection, we conduct extensive experiments on the “DrugMetaboliteAtlas” dataset. Table 2 shows the performance of various algorithms in terms of AUC, AUPR, and F1. In Table 2, the highest score in each column is shown in bold typeface.

**TABLE 2:** The best performance of all methods on the “DrugMetaboliteAtlas” dataset.

	**AUC**	**AUPR**	**F1**
DTInet	0.7430	0.2176	0.2951
IMCMDA	0.7913	0.3655	0.4345
GRNMF	0.9272	0.5847	0.5767
ILMF^–^	0.9223	0.5429	0.5662
ILMF	0.9402	0.6303	0.6052

*To ensure a fair comparison, the optimal parameters are selected from the ranges provided by these corresponding studies. For ILMF^–^ and ILMF, the above results are obtained when *c* = 2, ϕ = 1, λ = 8, and _*r=12*_.*

As shown in Table 2, ILMF achieves the best performance in terms of AUC, AUPR, and F1 on the “DrugMetaboliteAtlas” dataset. Specifically, compared with the second-best GRNMF algorithm, the performance of ILMF increases by 1.40, 7.80, and 4.94% in terms of AUC, AUPR, and F1, respectively. Additionally, the prediction performance of DTInet and IMCMDA is not satisfactory. We can observe from Table 2 that ILMF outperforms IMCMDA 18.82, 72.45, and 39.29% in AUC, AUPR, and F1, respectively. One possible reason is that IMCMDA does not take advantage of the local geometrical structure that resided within the original data. For GRNMF, it does not consider the important level parameter *c*, for simplicity, it views the known drug-metabolite pairs and the unobserved drug-metabolite pairs as equally important in predicting the latent associations between drugs and metabolites.

By comparing ILMF and ILMF^–^, we can also further verify the benefits of using the Vicus matrices of drugs and metabolites, indicating that exploiting the local structure information of drugs and metabolites could improve the performance for drug-metabolite association prediction.

### Parameter Analysis

There are several parameters in ILMF that need to be tuned: the important level parameter *c*, the dimensionality *k*_1_, *k*_2_ and *r* of projection matrices *W* and *H*, the regularization parameters λ and ϕ. For simplicity, we set *k*_1_ = 12 and *k*_2_ = 45 empirically. We adopted a grid search strategy to select the optimal combination from fixed ranges of λ and ϕ. In this study, we let λ and ϕ vary in the range {2^−3^,2^−2^,2^−1^,2^0^,2^1^,2^2^,2^3^}, *r* varies in the range {5,6,7,8,9,10,11,12} and *c* varies in the range {2,3,4,5,6,7,8}. We then conducted fivefold CV to evaluate the performance of ILMF under the combination of different parameters.

**FIGURE 2:**
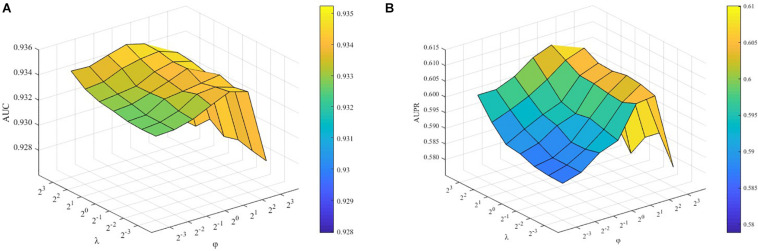
Performance of ILMF on “DrugMetaboliteAtlas” dataset with different values of λ and ϕ. **(A)** AUC versus λ and ϕ; **(B)** AUPR versus λ and ϕ.

To demonstrate how λ and ϕ affect the performance of the proposed ILMF, we fix other parameters and change the values of λ and ϕ, respectively. The AUC and AUPR scores are shown in Figures 2A,B with respect to different combinations of λ and ϕ.

_λ_ and ϕ are the parameters controlling the influence of feature regularization and graph regularization. As Figure 2 shows, when we fix the values of λ and increase the values of ϕ, the AUC scores increase initially and decrease after achieving the highest performance. These results demonstrate the advantages of introducing two kinds of regularization terms.

**FIGURE 3:**
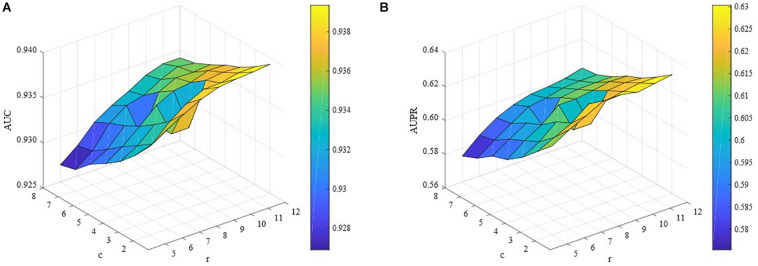
Performance of ILMF on “DrugMetaboliteAtlas” dataset with different values of *c* and *r*. **(A)** AUC versus *c* and *r*; **(B)** AUPR versus *c* and *r*.

In this study, we also conducted extensive experiments to demonstrate how *c* and *r* affect the performance of ILMF. We changed the values of *c* and *r* in the corresponding ranges with other parameters fixed. The AUC and AUPR scores are shown in Figures 3A,B with respect to different combinations of *c* and *r.* We can observe from Figure 3 that for a fixed value of *c*, the AUC scores increase as the values of *r* increase. However, when we fix the values of *r* and increase the values of *c*, the AUC scores decrease. Similar properties can be seen in terms of AUPR. This illustrates the importance and necessity of introducing levels of importance, which are assigned to the observed drug-metabolite interaction pairs.

## Predicting Novel Drug-Metabolite Associations

In this section, we evaluate the prediction ability of ILMF in identifying novel drug-metabolite associations. In our experiments, the entire dataset is used to train the ILMF model, and the optimal parameters are used to make a prediction. The unknown drug-metabolite interaction pairs are ranked based on the predicted association scores.

Table 3 shows the top 20 novel associations predicted by ILMF on the “DrugMetaboliteAtlas” dataset. In this table, the fourth column shows the predicted interaction probabilities of novel drug-metabolite pairs. For each pair, we retrieval the possible interaction from HMDB, DrugBank and other databases that may contain it, and list the corresponding ATC/drug names in the last column of Table 3. Since only a few databases include drug-metabolite association information, the fraction of new drug-metabolite interactions correctly predicted by ILMF may increase in the future. These promising results, which indicate that ILMF can successfully identify many novel associations, demonstrates that it is effective in predicting latent drug-metabolite associations from a sparse binary matrix.

**TABLE 3:** Top 20 novel associations predicted by ILMF on the “DrugMetaboliteAtlas” dataset.

**Rank**	**Drug category**	**Metabolite**	**Score**	**Evidence (ATC/drug name)**
1	C_HMG CoA reductase inhibitors-hydrophilic statin	TotPG	0.9915	C10AA03 (pravastatin)
2	M_Preparations inhibiting uric acid production	L.VLDL.FC	0.9891	M04AA01 (allopurinol)
3	M_Preparations inhibiting uric acid production	L.VLDL.P	0.9881	Unconfirmed
4	N_Benzodiazepine derivatives	UnsatDeg	0.9755	N03AE01 (clonazepam)
5	C_Angiotensin II antagonists-plain	XS.VLDL.FC	0.9687	Unconfirmed
6	C_Low-ceiling diuretics	XL.HDL.FC	0.9625	C03AA04 (chlorothiazide)
7	C_Low-ceiling diuretics	L.HDL.P	0.9588	C03AA03 (hydrochlorothiazide)
8	C_Low-ceiling diuretics	L.HDL.PL	0.9553	Unconfirmed
9	A_Insulins and analogs-fast-acting	FALen	0.9525	A10AB019 (insulin)
10	C_Low-ceiling diuretics	HDL.C	0.9493	Unconfirmed
11	B_Carbasalate calcium	ApoB	0.9419	Unconfirmed
12	C_Low-ceiling diuretics	HDL2.C	0.9346	Unconfirmed
13	C_Low-ceiling diuretics	UnsatDeg	0.9334	C03AA03 (hydrochlorothiazide)
14	C_Digoxin	S.VLDL.PL	0.9247	Unconfirmed
15	C_ACE inhibitors-plain	M.HDL.C	0.9240	C09AA01 (captopril)
16	C_HMG CoA reductase inhibitors-hydrophilic statin	S.HDL.CE	0.9219	C10AA03 (pravastatin)
17	C_Angiotensin II antagonists-plain	L.HDL.TG	0.9212	Unconfirmed
18	C_Fibrates	VLDL.D	0.9192	Unconfirmed
19	C_Angiotensin II antagonists-plain	PUFA	0.9188	C09CA01-08
20	M_Preparations inhibiting uric acid production	XL.VLDL.PL	0.9158	Unconfirmed

*TotPG, total phosphoglycerides; L.VLDL.P, concentration of large VLDL particles; L.VLDL.FC, free cholesterol in very large VLDL; UnsatDeg, estimated degree of unsaturation; XS.VLDL.FC, free cholesterol in very small VLDL; XL.HDL.FC, free cholesterol in very large HDL; L.HDL.P, concentration of large HDL particles; L.HDL.PL, phospholipids in large HDL; FALen, estimated description of fatty acid chain length- not actual carbon number; HDL.C, total cholesterol in HDL; ApoB, apolipoprotein B; HDL2.C, total cholesterol in HDL2; S.VLDL.PL, phospholipids in small VLDL; M.HDL.C, total cholesterol in medium HDL; S.HDL.CE, cholesterol esters in small HDL; L.HDL.TG, triglycerides in large HDL; VLDL.D, mean diameter for VLDL particles; PUFA, polyunsaturated fatty acids; XL.VLDL.PL, phospholipids in very large VLDL; VLDL, very-low-density lipoprotein; HDL, high-density lipoprotein.*

Note that the proposed ILMF is also effective when a new drug (or metabolite) without any known related metabolites (or drugs) is given. Once we have obtained the low-dimensional matrix representation $F_{n\,e\,w\,\left(i\right)}^{d}$ of a new drug or $F_{n\,e\,w\,\left(j\right)}^{d}$ of a metabolite, the interaction scores with known drugs or metabolites can be calculated by Eq. 15.

**FIGURE 4:**
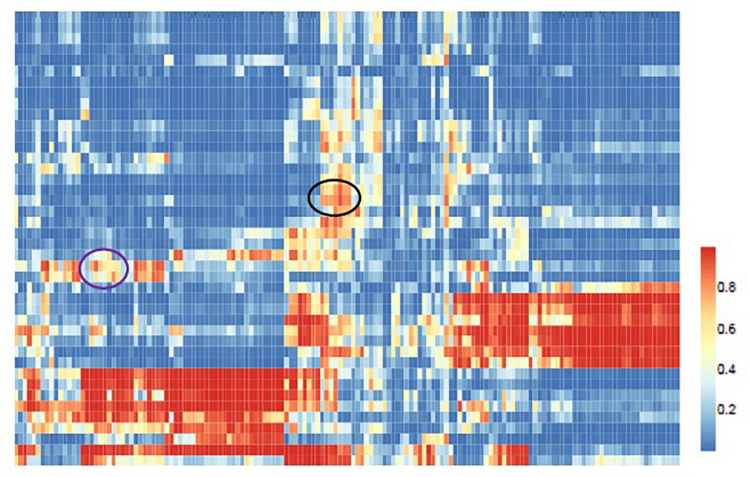
Global view of the predicted drug-metabolite associations. Hierarchical clustering of the ILMF scores between 42 drugs and 150 metabolites. The color of each cell represents the ILMF score of a drug (row) and a metabolite (column), where red/blue indicates high/low ILMF scores.

We further apply ILMF to detect the relationships between drugs and metabolites from a global view. ILMF is used to infer the metabolic potential of 42 drugs and chart the metabolic landscape of common drugs. First, we obtain a score matrix by applying ILMF on the whole “DrugMetaboliteAtlas” dataset. Then, hierarchical clustering is performed to explore the unknown relationships between drugs and metabolites (Figure 4). The scores indicate the interaction relationships between drugs and metabolites based on metabolic mechanisms. Therefore, the drugs and metabolites that are grouped may share metabolic overlaps in terms of pathways or microbial metabolites association profiles.

**FIGURE 5:**
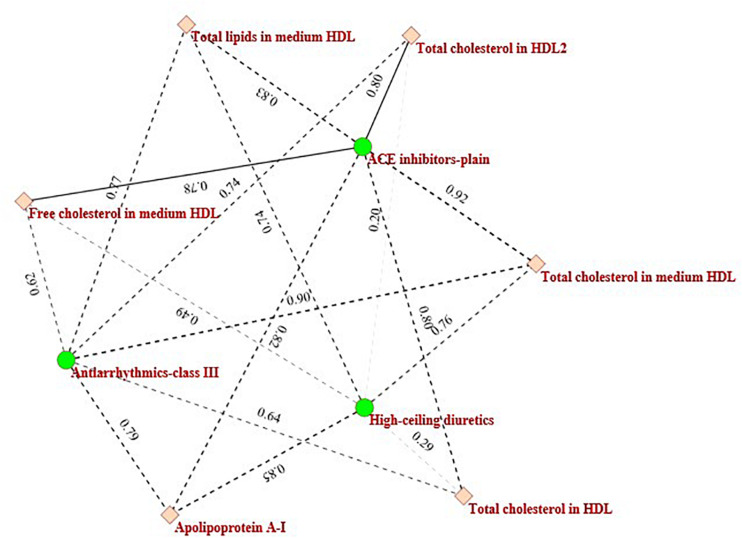
The sub-network consists of three drugs and six metabolites.

**FIGURE 6:**
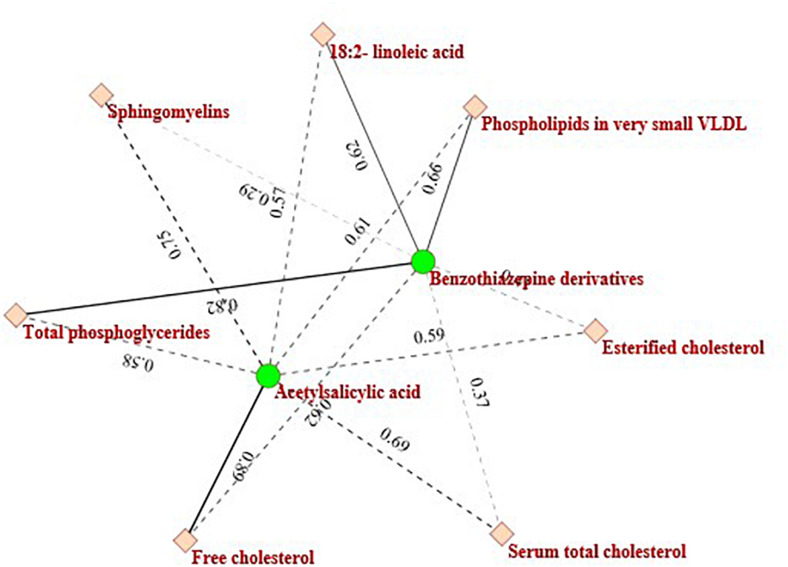
The sub-network consists of two drugs and seven metabolites.

In Figure 4 the black circled region shows a module that consists of three categories of drugs (*Antiarrhythmics-class III, ACE inhibitors-plain*, and *High-ceiling diuretics*) and six kinds of metabolites (*Total cholesterol in HDL2, Total cholesterol in HDL, Free cholesterol in medium HDL, Total cholesterol in medium HDL, Total lipids in medium HDL*, *and Apolipoprotein A-I*). These drugs and metabolites, which have no associations in the original drug-metabolite association matrix are identified by the proposed ILMF. The relationships between these drugs and metabolites have been reported in some literature. Figure 5 shows the connectivity of this module by extracting the corresponding rows and columns from the predicted drug-metabolite scoring matrix. The green circle denotes the three drugs mentioned above. The pink diamond denotes six metabolites. Solid lines indicate the true associations between drugs and metabolites. Dot lines indicate the predicted associations by ILMF. The values on the lines are the predicted scores. The bigger the score, the more trustworthy the predicted drug-metabolite interaction pair. This setting is also applied to Figure 6.

As shown in Figure 5, *Total cholesterol in medium HDL* is highly related to *Antiarrhythmics-class III* and *ACE inhibitors-plain* and the predicted interaction scores between them are 0.90 and 0.92, respectively. This is consistent with the fact that high *Total cholesterol* level usually leads to other complications, including diabetes, hyperlipidemia, hypertension, hypothyroidism, choledochus obstruction, coronary heart disease, atherosclerosis, and so on (Nelson, 2013). Miyazaki et al. (1999) also reported that ACE activity was significantly increased in the aorta of cholesterol-fed monkeys.

Another example is the purple circled region, which contains two kinds of drugs (Antithrombotic agents-Acetylsalicylic acid: B01AC06 and Benzothiazepine derivatives: C08DB01) and seven metabolites (Sphingomyelins, Serum total cholesterol, Total phosphoglycerides, Esterified cholesterol, Free cholesterol, 18:2-linoleic acid, and Phospholipids in very small VLDL). The drugs and metabolites in this module are also clinically relevant. Figure 6 describes the heterogeneous interaction network of this module. As Figure 6 indicates, Acetylsalicylic acid is related to Sphingomyelins (interaction probability is 0.7531). This finding is also consistent with another previous report by Suwalsky et al. (2013).

There have also been other biologically meaningful modules detected by ILMF. In short, the two examples mentioned above show the potential of the proposed ILMF algorithm in identifying the unknown associations between drugs and metabolites, which further demonstrates its effectiveness and efficiency.

## Conclusion

In this article, we propose a novel drug-metabolite association prediction method, named ILMF. ILMF could not only combine multiple-source drug–drug interaction, metabolite–metabolite interaction, and drug-metabolite association information into this framework but also take full advantage of the local geometrical structure inherent in the original data to improve prediction performance. In addition, we also exploited inductive matrix completion to guide the learning of projection matrices *U*, *V* based on the low-dimensional feature matrix of drugs (or metabolites) obtained from external data sources. The experimental results for the “DrugMetaboliteAtlas” dataset demonstrate the effectiveness of the proposed ILMF in predicting potential drug-metabolite associations. Moreover, in the last section of this study, we examine case studies on predicting novel drug-metabolite associations, the results of which may provide some valuable clues to biologists or clinicians.

Despite these promising findings, there are still some limitations to this proposed ILMF model. While fusing multiple types of biological data, the chemical structure information of drugs or metabolites is missing due to the fact that the initial “DrugMetaboliteAtlas” dataset only contains vague categories, particularly for metabolites. The low-dimension feature representation learning algorithm (clusDCA) is replaceable. More effective graph representation learning frameworks, such as graph convolution network (GCN), are expected to be combined with the ILMF framework to more accurately predict drug-metabolite associations. Lastly, the predicted drug-metabolite interactions need to be further validated in practice.

In the future, we will focus on developing new methods to explore the complex relationships between drugs and microbes, including the influence of microbes on drug activity or toxicity and so on.

## Data Availability Statement

Publicly available datasets were analyzed in this study. This data can be found here: https://github.com/chonghua-1983/ILMF.

## Author Contributions

YYM wrote the manuscript and developed the algorithms. YYM and LL developed the concept for the structure and content of the manuscript. QC wrote the code used in the manuscript. YJM critically revised the final manuscript. All authors reviewed and approved the final version of the manuscript.

## Conflict of Interest

The authors declare that the research was conducted in the absence of any commercial or financial relationships that could be construed as a potential conflict of interest.

## Footnotes

1 http://bbmri.researchlumc.nl/atlas/
